# AI-Driven Clinical Decision Support Systems: An Ongoing Pursuit of Potential

**DOI:** 10.7759/cureus.57728

**Published:** 2024-04-06

**Authors:** Malek Elhaddad, Sara Hamam

**Affiliations:** 1 Medicine, The Hospital for Sick Children, Toronto, CAN; 2 Medicine, Upper Canada College, Toronto, CAN; 3 Ophthalmology, Queen Elizabeth University Hospital, Glasgow, GBR

**Keywords:** ai bias, interpretability, user-centric interface, deep learning models, natural language processing (nlp), convolutional neural networks (cnn), recurrent neural networks, machine learning algorithms, artificial intelligence (ai), clinical decision support systems (cdss)

## Abstract

Clinical Decision Support Systems (CDSS) are essential tools in contemporary healthcare, enhancing clinicians' decisions and patient outcomes. The integration of artificial intelligence (AI) is now revolutionizing CDSS even further. This review delves into AI technologies transforming CDSS, their applications in healthcare decision-making, associated challenges, and the potential trajectory toward fully realizing AI-CDSS's potential. The review begins by laying the groundwork with a definition of CDSS and its function within the healthcare field. It then highlights the increasingly significant role that AI is playing in enhancing CDSS effectiveness and efficiency, underlining its evolving prominence in shaping healthcare practices. It examines the integration of AI technologies into CDSS, including machine learning algorithms like neural networks and decision trees, natural language processing, and deep learning. It also addresses the challenges associated with AI integration, such as interpretability and bias. We then shift to AI applications within CDSS, with real-life examples of AI-driven diagnostics, personalized treatment recommendations, risk prediction, early intervention, and AI-assisted clinical documentation. The review emphasizes user-centered design in AI-CDSS integration, addressing usability, trust, workflow, and ethical and legal considerations. It acknowledges prevailing obstacles and suggests strategies for successful AI-CDSS adoption, highlighting the need for workflow alignment and interdisciplinary collaboration. The review concludes by summarizing key findings, underscoring AI's transformative potential in CDSS, and advocating for continued research and innovation. It emphasizes the need for collaborative efforts to realize a future where AI-powered CDSS optimizes healthcare delivery and improves patient outcomes.

## Introduction and background

From data to decisions: the influence of CDSS in clinical practice

Imagine having a virtual assistant right by your side, equipped with the latest medical knowledge and ready to help you make the best decisions for your patients. That's precisely what Clinical Decision Support Systems (CDSS), interpreted as the computer programs that assist healthcare professionals in making medical decisions, represent: a pivotal advancement in healthcare, integrating technological prowess with medical expertise to augment clinical decision-making processes. These systems are designed to provide healthcare professionals with actionable insights, evidence-based recommendations, and patient-specific information at the point of care, thereby enhancing diagnostic accuracy, treatment efficacy, and patient outcomes. CDSS operates on a foundation of medical knowledge, algorithms, and patient data, aiming to bridge the gap between vast medical information and timely, informed clinical decisions. At its core, CDSS serves as a cognitive aid, assisting clinicians in navigating the complexities of modern medicine. It analyzes patient data, medical literature, and best practices to offer tailored suggestions, reminders, and alerts to healthcare providers during the care delivery process. The significance of CDSS lies in its potential to mitigate diagnostic errors, optimize treatment plans, reduce healthcare costs, and ultimately, improve patient safety and quality of care. According to the Agency for Healthcare Research and Quality (AHRQ), CDSS interventions have been shown to enhance healthcare quality by facilitating adherence to clinical guidelines, reducing medication errors, and minimizing adverse drug events [1]. Moreover, Chen et al. emphasized the versatility and widespread applicability of CDSS, demonstrating its utility across various healthcare settings, ranging from primary care clinics to intensive care units [2].

Empowering CDSS: the growing role of AI

In recent years, the integration of artificial intelligence (AI) has propelled CDSS into a new era of sophistication and effectiveness. AI technologies, including machine learning, natural language processing (NLP), and deep learning, have revolutionized the capabilities of CDSS, enabling it to process and interpret vast amounts of healthcare data with unprecedented speed and accuracy. Machine learning algorithms, a branch of AI that teaches computers to learn from data and improve their performance over time without being explicitly programmed, such as neural networks and decision trees, empower CDSS to discern patterns, recognize correlations, and derive insights from complex datasets [3]. These algorithms continuously learn from new data inputs, refining their predictive capabilities and adapting to evolving clinical scenarios. Consequently, CDSS powered by machine learning can offer personalized recommendations tailored to individual patient needs, enhancing clinical decision-making and patient outcomes [4]. Likewise, NLP, another subset of AI that focuses on enabling computers to understand, interpret, and generate human language in a way that is meaningful and useful, has emerged as a cornerstone of AI-driven CDSS, enabling the extraction and analysis of valuable information from unstructured clinical text, including electronic health records (EHRs), medical notes, and research literature. By transforming free-text data into structured formats, NLP facilitates semantic understanding, information retrieval, and knowledge extraction, empowering CDSS to derive actionable insights from diverse sources of clinical information. Another AI technology that represents a move up in this contest is the deep learning models, a subset of machine learning that involves neural networks with multiple layers, hence the term "deep". Deep learning models, characterized by their multi-layered neural architectures, have further expanded the capabilities of AI-driven CDSS, particularly in image analysis, pattern recognition, and predictive modeling [3,5]. In their research focused on Dermatologist-level classification of skin cancer, Esteva et al. showed that deep convolutional neural networks (CNNs) achieve performance on par with all tested experts, demonstrating an AI capable of classifying skin cancer with a level of competence comparable to dermatologists [6].

The purpose of this review article is to explore the role and potential of AI in transforming CDSS. By examining current research, practical applications, and emerging trends, this article aims to elucidate the symbiotic relationship between AI technologies and CDSS, delineating their collective impact on healthcare delivery, clinical decision-making, and patient outcomes. Through an in-depth analysis of AI technologies, applications, human-centered considerations, challenges, and future directions, this review seeks to inform healthcare professionals, researchers, policymakers, and stakeholders about the transformative possibilities and complexities inherent in the convergence of AI and CDSS. In essence, this review serves as a roadmap for understanding the past, present, and future trajectory of AI-driven CDSS, illuminating the opportunities, challenges, and ethical considerations that accompany this paradigm shift in healthcare delivery and decision support.

## Review

An overview of AI technologies within CDSS

AI has recently emerged as a transformative force in healthcare, particularly in the realm of CDSS. Leveraging advanced computational techniques, AI augments healthcare professionals' decision-making processes, thereby enhancing patient care delivery and outcomes. To begin with, we will briefly outline the three key AI technologies propelling innovation within CDSS, delineating their unique characteristics before proceeding to explore each in greater depth: machine learning algorithms, NLP, and deep learning models. Think of them as super smart assistants that crunch vast amounts of patient data to predict future health issues and suggest the best course of action, Machine learning algorithms empower CDSS to analyze patient data and make predictions, aiding in diagnosis and treatment planning. NLP allows CDSS to extract insights from clinical text, streamlining documentation and facilitating data retrieval. Deep learning models, an even more advanced form of machine learning that excels at finding complex patterns, automate complex pattern extraction from diverse medical data, including images and sequential data like ECGs, enhancing diagnostic accuracy and personalized patient care. Each of these technologies brings unique capabilities, from predictive analytics with machine learning to text analysis with NLP and intricate data pattern recognition with deep learning, all aimed at improving healthcare decision-making and patient outcomes.

AI Technologies Within CDSS: Machine Learning Algorithms

Machine learning algorithms serve as the cornerstone of modern CDSS, empowering healthcare providers with predictive analytics capabilities and decision-making support. These algorithms enable CDSS to process and analyze vast volumes of patient data, including EHRs, medical imaging, and genomic information, to extract meaningful insights and inform clinical decisions. Neural Networks, Decision Tree algorithms, Support Vector Machines (SVMs), Bayesian Networks, and Ensemble Learning Methods represent distinct classes of machine learning algorithms. While each falls under the broader category of machine learning, they employ unique methodologies and techniques for learning from data and making predictions. Neural networks, a class of machine learning models inspired by the structure and functioning of the human brain, have gained prominence in CDSS for their ability to learn complex patterns from data. CNNs, in particular, excel in medical image analysis tasks, such as identifying abnormalities in radiological scans and detecting lesions in histopathological images. Studies have demonstrated the efficacy of CNN-based CDSS in improving diagnostic accuracy and facilitating early disease detection [5,6]. Decision Tree (DT) algorithms represent another class of machine learning techniques widely employed in CDSS. These algorithms construct a tree-like structure to model decision paths based on input variables, enabling healthcare providers to navigate complex clinical scenarios and formulate personalized treatment plans. Random Forests and Gradient Boosting Machines are popular DT algorithms known for their robustness and predictive performance in healthcare applications [7]. Moreover, Support Vector Machines (SVMs), Bayesian Networks, and ensemble learning methods are among the diverse repertoire of machine learning algorithms utilized in CDSS to address various clinical challenges, including disease diagnosis, risk stratification, and treatment optimization [8]. By harnessing the power of machine learning, CDSS can leverage historical patient data to anticipate future health outcomes and tailor interventions to individual patient needs.

AI Technologies Within CDSS: NLP

NLP, on the other hand, plays a pivotal role in unlocking insights from unstructured clinical text, such as physician notes, discharge summaries, and medical literature. With the proliferation of EHRs and digital health platforms, healthcare organizations are inundated with textual data, presenting both opportunities and challenges for leveraging this information effectively. NLP algorithms enable CDSS to parse, interpret, and extract relevant information from clinical narratives, facilitating automated clinical documentation, coding, and information retrieval. These algorithms leverage linguistic analysis techniques to identify clinical concepts, extract structured data elements, and populate structured databases, thereby streamlining clinical workflows and enhancing data interoperability [9]. As one of the key NLP tasks, sentiment analysis refers to the computational process of discerning and analyzing the emotional nuances expressed within textual data. In essence, it involves the systematic examination of text to determine the underlying sentiments or attitudes conveyed by the author, or speaker. This can encompass a spectrum of emotions, including but not limited to positive, negative, or neutral sentiments. Sentiment analysis algorithms often utilize various techniques, such as machine learning and linguistic analysis, to identify and categorize the emotional tone of the text accurately. It plays a crucial role in NLP by enabling the automated extraction and interpretation of emotional content from textual data, thereby facilitating a deeper understanding of human communication and behavior. On the other hand, there exists another pivotal NLP task known as entity recognition, which serves to identify and categorize specific entities such as names, dates, and medical terminology within the textual corpus. Information extraction, meanwhile, is centered on the systematic retrieval of structured data from unstructured text, facilitating the extraction of vital clinical facts and treatment plans. These fundamental NLP tasks collectively equip CDSS with the capability to meticulously analyze textual data, thus empowering healthcare professionals with actionable insights essential for informed decision-making. By analyzing the sentiment and tone of clinical narratives, NLP algorithms can discern patient attitudes, preferences, and treatment adherence patterns, thereby informing personalized care plans and patient engagement strategies.

AI Technologies Within CDSS: Deep Learning

Finally, deep learning, a subset of machine learning, has revolutionized CDSS by enabling the automatic extraction of complex patterns and representations from heterogeneous medical data sources. Unlike traditional machine learning algorithms, which require handcrafted features and domain-specific knowledge, deep learning models learn hierarchical representations of data through multiple layers of interconnected neurons, enabling them to capture intricate relationships and nuances in medical datasets [10]. CNNs, originally developed for image processing tasks, have been adapted for various medical imaging applications, including lesion detection, organ segmentation, and disease classification [6]. By leveraging hierarchical feature extraction and spatial hierarchies, CNN-based CDSS can utilize advanced computational techniques to analyze medical images in-depth. These systems are adept at detecting nuanced abnormalities within the images, demonstrating both high sensitivity and specificity in their diagnostic capabilities. The hierarchical feature extraction process involves the identification of various image features at multiple levels of abstraction, enabling the system to discern subtle details indicative of abnormalities. Additionally, spatial hierarchies are leveraged to understand the spatial relationships between different image features, further enhancing the system's ability to accurately interpret complex medical images. By employing these sophisticated techniques, CNN-based CDSS systems serve as invaluable tools for radiologists and clinicians, providing them with enhanced support in the diagnostic decision-making process. Their capability to identify subtle abnormalities with precision aids in early detection and accurate diagnosis, ultimately leading to improved patient outcomes. Similarly, Recurrent Neural Networks (RNNs) and their variants, such as long short-term memory (LSTM) networks, excel in processing sequential data modalities, such as electrocardiograms (ECGs), electroencephalograms (EEGs), and clinical time-series data. Choi et al. and Lakhani and Sundaram have both highlighted the capabilities of RNN-based CDSS being able to model temporal dependencies, detect anomalous patterns, and predict clinical outcomes, thereby facilitating early intervention and personalized patient care [11,12]. The potential of deep learning models in CDSS extends beyond medical imaging and time-series data analysis to encompass clinical NLP, genomics, and personalized medicine. By integrating multimodal data sources and leveraging advanced neural network architectures, CDSS can provide holistic insights into patient health status, facilitate early disease detection, and optimize treatment strategies tailored to individual patient profiles.

Brief Overview of Challenges in AI Technologies Integration With CDSS

While these AI technologies hold immense promise for transforming CDSS and improving healthcare delivery, their integration into clinical practice is fraught with challenges that must be addressed to ensure their effectiveness, safety, and ethical use. These challenges include technical limitations, regulatory constraints, ethical considerations, and organizational barriers that impact the adoption and scalability of AI-driven CDSS. One of the main challenges associated with AI integration in CDSS is the interpretability and transparency of AI models. For example, deep learning models, characterized by their complex architectures and black-box nature, pose challenges for clinicians and healthcare providers seeking to understand the rationale behind AI-driven recommendations and predictions. Moreover, the generalizability and robustness of many AI models across diverse patient populations, clinical settings, and data modalities represent critical challenges in CDSS. AI algorithms trained on biased or unrepresentative datasets may exhibit performance disparities across demographic groups or clinical subpopulations, leading to disparities in healthcare outcomes and exacerbating existing health inequities. In their detailed examination of racial bias in an algorithm used to manage the health of populations, Obermeyer et al. underscored the necessity of mitigating bias, ensuring algorithmic fairness, and promoting inclusivity in AI-driven CDSS [13]. The achievement of these goals is contingent upon rigorous data collection, validation, and algorithmic auditing processes. Furthermore, regulatory constraints and privacy concerns pose significant hurdles to the widespread adoption of AI-driven CDSS in healthcare. Compliance with regulatory frameworks, such as the Health Insurance Portability and Accountability Act (HIPAA) in the United States or the General Data Protection Regulation (GDPR) in the European Union, is essential to safeguard patient privacy, confidentiality, and data security. Additionally, navigating the regulatory landscape governing medical device approvals, software validation, and CDSS poses challenges for healthcare organizations and technology vendors seeking to deploy AI-driven solutions in clinical practice [14].

An overview of AI applications within CDSS

The integration of AI has significantly reshaped CDSS, offering innovative solutions that optimize patient care and streamline clinical workflows. Through the application of advanced algorithms and machine learning techniques, AI-driven CDSS empowers healthcare professionals by providing invaluable insights and assistance across various facets of medical practice. From diagnostic support to personalized treatment recommendations, proactive risk prediction, and the facilitation of clinical documentation, AI in CDSS revolutionizes traditional approaches, paving the way for a new era of precision medicine as will be examined hereafter in more detail.

AI Applications Within CDSS: Diagnostic Support

One of the most impactful applications of AI-driven CDSS is in providing diagnostic support. AI algorithms, particularly deep learning models, have shown remarkable capabilities in analyzing medical images, such as X-rays, MRIs, and histopathology slides, thereby assisting clinicians in making accurate diagnoses. For instance, Esteva et al. showcased the potential of deep neural networks in dermatology diagnostics by achieving dermatologist-level classification of skin cancer from dermoscopic images [6]. Their study, which was published in Nature Medicine, highlighted the effectiveness of AI in enhancing diagnostic accuracy and improving patient outcomes. Furthermore, NLP techniques enable AI systems to extract valuable insights from unstructured clinical notes, thereby facilitating a comprehensive understanding of patient conditions. For instance, Gholipour et al. demonstrated the utility of NLP in identifying cancer concepts by extracting relevant information from clinical notes [15]. Their findings, published in a systematic review, underscore the significant role of AI in augmenting diagnostic precision through text analysis. This review systematically examined studies that used NLP methods to identify cancer concepts from clinical notes automatically, highlighting the potential of AI in the field of oncology [15].

AI Applications Within CDSS: Personalized and Proactive Healthcare

AI-CDSS extends beyond simply analyzing data support in the diagnosis, but it also reveals its potential for personalized treatment recommendations tailored to individual patient characteristics. This revolutionizes traditional approaches to healthcare delivery by leveraging a holistic view of each patient. By analyzing diverse patient data, including genetic profiles, medical histories, and treatment outcomes, AI algorithms can identify optimal treatment strategies that optimize efficacy and minimize risks. Komorowski et al. introduced a reinforcement learning algorithm, AI clinician, for personalized sepsis treatment, as published in Nature Biomedical Engineering [16]. This algorithm continuously learns from patient data and treatment responses to dynamically adjust therapeutic regimens, resulting in improved patient outcomes compared to standard protocols. Moreover, predictive modeling powered by AI enables proactive healthcare management by identifying patients at high risk of developing specific conditions. Choi et al. demonstrated the use of RNN models, Doctor AI, for early detection of heart failure onset [11]. Their study, published in the Journal of the American Medical Informatics Association, exemplifies AI's role in pre-emptive interventions to mitigate risks and improve patient outcomes.

AI Applications Within CDSS: Risk Prediction and Early Intervention

Another notable and increasingly successful application is the proficiency of AI-driven CDSS in risk prediction and early intervention, facilitating clinicians in foreseeing and addressing potential health threats before they develop into severe conditions. By leveraging machine learning algorithms and real-time patient data, these systems stratify patients based on their risk profiles, allowing for targeted interventions and resource allocation. Deep learning models have been instrumental in predicting cardiovascular events in patients with diabetes, as demonstrated by Choi et al. [11]. Their study showcases AI's ability to analyze EHRs and accurately identify individuals at high risk of developing cardiovascular complications, enabling timely interventions to prevent adverse outcomes. Similarly, AI-driven CDSS can facilitate the early detection of diseases through the recognition of subtle patterns indicative of pathological processes. Ryu et al. developed a CNN model capable of predicting diabetic retinopathy from OCTA images with an accuracy of 91%-98%, underscoring AI’s potential in facilitating timely interventions to prevent irreversible damage and improve patient outcomes [17].

AI Applications Within CDSS: Clinical Documentation

Last in this list, but not covering everything, is clinical documentation, a critical yet time-consuming aspect of healthcare delivery, often burdening clinicians with extensive paperwork and documentation tasks. AI technologies offer solutions to streamline documentation processes, allowing clinicians to focus more on patient care. NLP algorithms automate clinical documentation by extracting relevant information from unstructured clinical notes and populating EHRs with structured data. Nuthakki et al. evaluated the use of NLP for automating clinical documentation in emergency department notes, demonstrating significant time savings for clinicians without compromising documentation quality [18]. Furthermore, AI-driven CDSS integrates voice recognition and natural language understanding capabilities to enable real-time documentation during patient encounters. By seamlessly integrating documentation tasks into clinical workflows, these systems enhance efficiency and accuracy while improving the overall quality of patient care.

Undeniably, the breadth and depth of AI-driven CDSS is vast, extending well beyond the few applications touched upon in this review and spanning a broad spectrum within the healthcare sector. These applications are not merely expansive, but transformative, fundamentally redefining the landscape of clinical and medical practice. They have ushered in an era of unparalleled efficiencies, markedly enhancing workflow productivity, liberating clinicians to devote more time to patient care, augmenting diagnostic precision, and mitigating potential errors. This ultimately culminates in improved patient outcomes. This paradigm shift signals the dawn of a new epoch in precision medicine, a testament to the transformative power of AI in healthcare.

Beyond technology: exploring human-AI interaction in CDSS

Given the earlier, it is crucial to ask if the integration of AI within CDSS is merely a blend of algorithms and machines, or does it go deeper, intertwining with the intricate dynamics of human interaction? The role of AI in healthcare transcends the boundaries of technology, intersecting with the human experience in different ways. This intersection is not a simple crossroad, but a complex interchange where clinicians leveraging AI-driven CDSS for decision-making meet patients benefiting from AI-enhanced diagnostics and treatments. The interface between AI and human factors within CDSS is not static, but an evolving landscape. Adding to this complexity are the legal and ethical considerations that accompany the implementation of AI in healthcare, layering further intricacy to this interplay. In the following, we explore the intricate interplay between AI technology and the human element in CDSS, examining its implications for clinical practice, patient care, and societal ethics. Amidst healthcare operations, particularly within AI-driven CDSS, the role of AI is not just about technological advancement but also about how it integrates with and impacts the human experience. Human-centered design (HCD) and evaluation are therefore considered integral aspects of ensuring that AI-driven CDSS not only function effectively but are also accepted and trusted by healthcare professionals and patients alike.

User-Centric AI-CDSS: User-Centered Design

User-centered design (UCD) is a fundamental principle that places the needs, preferences, and limitations of end-users at the forefront of system development. In the context of AI-CDSS, adopting a UCD approach is paramount to designing systems that are intuitive, efficient, and user-friendly for healthcare providers. By involving end-users, including clinicians, nurses, and other healthcare professionals, throughout the design and development process, AI-CDSS can better align with their workflow, preferences, and clinical decision-making needs. Involving end-users in the design process is crucial for ensuring that AI-CDSS meets their needs and preferences. By actively engaging clinicians and other stakeholders, we can create systems that enhance rather than disrupt clinical workflows [19]. Moreover, incorporating feedback from end-users through iterative design cycles enables developers to refine AI-CDSS iteratively, addressing usability issues, improving system performance, and enhancing user satisfaction. User-centric interface principles, such as simplicity, consistency, and feedback-driven iteration, guide the development of AI-CDSS interfaces and functionalities, fostering user acceptance and adoption in clinical settings.

User-Centric AI-CDSS: Clinician Experiences With AI-CDSS

On the other hand, another key factor in encouraging user acceptance and adoption is the importance of hearing feedback and understanding clinician experiences with AI-CDSS. This is essential for evaluating system usability, identifying workflow challenges, and assessing the impact on patient care delivery. Clinicians' perceptions, attitudes, and interactions with AI-driven tools influence their acceptance and utilization in real-world clinical practice. Therefore, conducting qualitative studies, surveys, and interviews with healthcare professionals can provide valuable insights into their experiences, challenges, and expectations regarding AI-CDSS adoption. Clinicians' experiences with AI-CDSS vary widely, influenced by factors such as system usability, perceived usefulness, and alignment with clinical workflows. By soliciting feedback from end-users, we can identify areas for improvement and enhance the user experience [20]. Clinician feedback can inform iterative refinements to AI-CDSS interfaces, algorithms, and decision support functionalities, ensuring that they meet the diverse needs and preferences of healthcare providers across different specialties and care settings. Moreover, fostering a culture of collaboration and co-creation between developers and end-users facilitates the development of AI-CDSS that are not only technologically robust but also clinically relevant and user-centric.

User-Centric AI-CDSS: Usability and Trust

That said, addressing critical factors such as usability, trust, and workflow considerations is therefore paramount to the effective adoption and utilization of AI-CDSS in clinical practice. Healthcare professionals rely on AI-driven tools to support their decision-making processes, streamline workflows, and improve patient outcomes. Therefore, ensuring that AI-CDSS is intuitive, reliable, and seamlessly integrated into existing clinical workflows is essential for their successful implementation and utilization. Usability is key to the adoption of AI-CDSS in clinical practice. Systems must be designed with the end-user in mind, taking into account their workflow, cognitive load, and interaction preferences to maximize usability and user satisfaction. Lastly, but perhaps most significantly, we arrive at the cornerstone of healthcare practitioners' acceptance and adoption: trust. Indeed, in the domain of health and clinical decision-making, trust stands as the bedrock upon which healthcare professionals rely. Establishing trust in AI-CDSS among healthcare providers requires transparency, rationality, and accountability in system design and operation. Thus, clinicians must actually understand how AI algorithms generate recommendations, the underlying evidence supporting those recommendations, and the limitations and uncertainties inherent in such AI-driven predictions. Providing clinicians with access to relevant clinical guidelines, decision support rationales, and performance metrics enhances trust in AI-CDSS and fosters their acceptance and adoption in clinical settings.

User-Centric AI-CDSS: Ethical and Legal Implications

However, is it all about the human? Not quite. As we delve deeper into healthcare technology, it always becomes evident that the societal perspective plays a crucial role in shaping the AI-CDSS impact. Beyond individual interactions, broader legal and ethical considerations loom large, shaping the landscape of healthcare innovation. As AI technologies continue to proliferate in healthcare, addressing ethical and legal implications is paramount to ensuring patient safety, privacy, and autonomy. AI-CDSS raises complex ethical dilemmas related to data privacy, algorithmic bias, transparency, accountability, and informed consent. Healthcare organizations must navigate regulatory frameworks, such as the Health Insurance Portability and Accountability Act (HIPAA) in the United States or the General Data Protection Regulation (GDPR) in the European Union, to safeguard patient data and ensure compliance with data protection laws. Ethical considerations are central to the responsible development and deployment of AI-CDSS. Healthcare organizations must prioritize patient privacy, autonomy, and safety, while also promoting equity, fairness, and transparency in AI-driven decision-making processes. Moreover, addressing algorithmic bias and fairness is essential for mitigating disparities in healthcare outcomes and ensuring equitable access to AI-driven decision support. Healthcare organizations must implement robust strategies for detecting, mitigating, and monitoring bias in AI algorithms, including data preprocessing techniques, algorithmic auditing, and bias-aware model evaluation methods. Additionally, fostering a culture of ethical awareness and accountability among developers, clinicians, and policymakers is critical for navigating the ethical complexities of AI-CDSS and promoting responsible innovation in healthcare. In conclusion, HCD and evaluation are essential for the successful integration of AI technologies into CDSS. By prioritizing user needs, incorporating clinician feedback, addressing usability, trust, and workflow considerations, and navigating ethical and legal implications, healthcare organizations can develop AI-CDSS that enhance clinical decision-making, improve patient outcomes, and promote the responsible and ethical use of AI in healthcare delivery [21-24].

The journey ahead: challenges and opportunities in AI-CDSS

In the ever-evolving landscape of healthcare, AI-CDSS now emerges as a beacon of hope, poised to redefine medical care delivery. Yet, amid its promising potential, lie multifaceted challenges that demand meticulous attention and strategic resolution. The journey towards harnessing the full potential of AI-CDSS is laden with technical intricacies, workflow intricacies, and societal perceptions, all of which necessitate comprehensive exploration and strategic navigation. In the following, we will briefly embark on a concise review of the challenges and opportunities entwined within the domain of AI-CDSS. From grappling with the technical limitations inherent in AI algorithms to orchestrating seamless integration within existing clinical workflows, each facet unveils its own complexities, beckoning for insightful elucidation. Moreover, the attitudinal barriers entrenched within the healthcare ecosystem demand profound consideration, emphasizing the indispensable role of cultural shifts and stakeholder buy-in in fostering sustainable AI-CDSS adoption. As we navigate through these challenges, we do not tread alone. Interdisciplinary collaboration emerges as a cornerstone, propelling us towards innovative solutions and shaping the trajectory of AI-driven healthcare. By fostering synergies across diverse domains - from computer science to clinical practice, from ethics to engineering - we can pave the way for a future where AI-CDSS not only augments clinical decision-making but also transcends boundaries, enriching the quality of care delivered to patients worldwide.

The Journey Ahead: Addressing Challenges

Despite the advancements in AI technologies, several technical limitations might always hinder the seamless integration of AI into CDSS. Interpretability of AI algorithms remains a significant concern, particularly in healthcare, where the decisions made by AI systems directly impact patient care. Deep learning models, while powerful in their predictive capabilities, often operate as black boxes, making it challenging for clinicians to understand the rationale behind AI-driven recommendations. Addressing the interpretability challenge requires the development of explainable AI techniques that elucidate how AI algorithms arrive at specific conclusions. Smith et al. discussed the challenge that comes with the interpretability of AI-CDSS for clinicians. They noted that while “explainability” is frequently offered as an important principle for good AI use, it is challenging to elicit an adequate level of explanation for the basis of outputs from those AI-CDSSs built on machine learning. They further highlighted that the reasoning behind AI-CDSS recommendations often remains opaque, raising questions about appropriate clinical use and suggesting a precautionary approach where only clinicians with proven knowledge of the clinical specialty in question are permitted to use AI recommendations [25,26]. Furthermore, bias in AI algorithms, as discussed earlier, still poses another significant challenge, potentially exacerbating disparities in healthcare delivery. Biases inherent in training data or algorithmic design can lead to erroneous predictions and contribute to inequities in patient outcomes. Mitigating such bias, as emphasized by Obermeyer et al. and Ferrara, requires diligent and continuous data curation, algorithmic auditing, and the implementation of fairness-aware machine learning techniques to ensure that AI-CDSS uphold principles of fairness and equity across diverse patient populations [13,27,28].

Another substantial obstacle that carries considerable significance is the reality that the successful integration of AI-CDSS into clinical workflows hinges on aligning technological advancements with the operational realities of healthcare settings. Resistance to change, lack of familiarity with AI technologies, and concerns regarding workflow disruption are common attitudinal barriers encountered during the adoption of AI-CDSS. Clinicians may perceive AI as a threat to their autonomy or professional judgment, leading to reluctance to embrace AI-driven decision-support tools. Addressing workflow alignment requires a nuanced understanding of the clinical environment and the unique challenges faced by healthcare providers. User-centric design, training programs, and change management initiatives can mitigate resistance and facilitate the seamless integration of AI-CDSS into clinical workflows [25,29]. Last, but certainly not least, data silos within healthcare systems present another significant challenge to the widespread implementation of AI-CDSS; Integrating AI-driven tools with existing EHR systems, medical devices, and clinical workflows necessitates standardized data formats, interoperability standards, and robust data governance frameworks. Overcoming these technical barriers necessitates collaboration between healthcare institutions, technology vendors, and regulatory bodies to establish interoperable infrastructure and promote data-sharing initiatives.

The Journey Ahead: Overcoming Challenges

To realize the full potential of AI in transforming healthcare delivery, stakeholders must employ multifaceted strategies to overcome existing challenges and facilitate the successful adoption of AI-CDSS. Education and training programs play a crucial role in familiarizing healthcare professionals with AI technologies, fostering digital literacy, and instilling confidence in utilizing AI-driven decision-support tools in clinical practice. Investing in education and training is crucial for preparing healthcare professionals for the AI-driven future of healthcare. This is supported by the study by Banerjee et al., which found that trainee doctors have an overall positive perception of AI technologies’ impact on clinical training and are optimistic about its potential to improve “research and quality improvement” skills and facilitate “curriculum mapping.” The study also recommends formalizing “Applied AI” topics in curricula and leveraging digital technologies to deliver clinical education. This aligns with the need for continuous learning programs, hands-on workshops, and interdisciplinary collaborations to empower clinicians to harness the full capabilities of AI-CDSS and leverage them to improve patient care outcomes [30]. Furthermore, interdisciplinary collaboration between clinicians, data scientists, engineers, and policymakers facilitate the co-creation of AI-driven solutions tailored to the unique needs and challenges of healthcare delivery. In line with this, numerous studies, including the research conducted by Sallam et al., have emphasized the growing need for rigorous and standardized evaluation approaches amidst the rapid development of AI technologies in the healthcare sector. These studies underscore the importance of criteria such as completeness, absence of false information, evidence support, appropriateness, and relevance in ensuring the effectiveness and reliability of AI-driven solutions [31]. By adhering to such criteria, interdisciplinary teams can develop AI-CDSS that are not only clinically relevant, ethically sound, and scalable across diverse healthcare settings but also rigorously evaluated to meet the highest standards of quality and reliability.

In essence, while AI holds immense promise for transforming CDSS, addressing the inherent challenges, and charting future directions require a concerted effort from stakeholders across the healthcare ecosystem. By acknowledging technical limitations, addressing workflow alignment and attitudinal barriers, implementing strategies for successful adoption, and embracing interdisciplinary collaboration, we can navigate the complexities of AI-CDSS and unlock its full potential in revolutionizing healthcare delivery [27].

## Conclusions

In the dynamic landscape of healthcare, the integration of AI with CDSS presents unparalleled opportunities for revolutionizing patient care, clinical outcomes, and innovation. This review delved into AI's pivotal role in reshaping CDSS, its underlying technologies, human-centered considerations, challenges, and future trajectories. AI, comprising machine learning algorithms, NLP, and deep learning models, empowers CDSS to quickly, and efficiently analyze intricate vast data, extract insights, and augment clinical decision-making across diverse domains. However, integrating AI into CDSS poses several challenges, including technical limitations, AI bias, and concerns about the interpretability of AI algorithms functioning as opaque black boxes, thereby impeding clinicians' comprehension of AI-driven recommendations. Addressing these challenges requires the development of explainable AI techniques to elucidate decision-making processes and the implementation of fairness-aware AI to mitigate bias. Furthermore, aligning AI-CDSS with clinical workflows and addressing attitudinal barriers among healthcare professionals is imperative for successful adoption. Finally, while education and training initiatives can enhance digital literacy among healthcare professionals, interdisciplinary collaboration is essential for fostering the development of clinically relevant and scalable AI-CDSS solutions. By acknowledging these challenges and deploying multifaceted strategies to address them, the broader healthcare community can effectively harness the potential of AI-CDSS to revolutionize healthcare delivery.
